# Loss of the Conserved Alveolate Kinase MAPK2 Decouples *Toxoplasma* Cell Growth from Cell Division

**DOI:** 10.1128/mBio.02517-20

**Published:** 2020-11-10

**Authors:** Xiaoyu Hu, William J. O’Shaughnessy, Tsebaot G. Beraki, Michael L. Reese

**Affiliations:** a Department of Pharmacology, University of Texas Southwestern Medical Center, Dallas, Texas, USA; b Department of Biochemistry, University of Texas Southwestern Medical Center, Dallas, Texas, USA; University of Pittsburgh

**Keywords:** kinase, centrosomes, organelles, MAP kinases, *Toxoplasma gondii*, apicomplexan parasites, apicoplast, cell cycle checkpoints, mitochondria

## Abstract

Mitogen-activated protein kinases (MAPKs) are a conserved family of protein kinases that regulate signal transduction, proliferation, and development throughout eukaryotes. The apicomplexan parasite Toxoplasma gondii expresses three MAPKs. Two of these, extracellular signal-regulated kinase 7 (ERK7) and MAPKL1, have been implicated in the regulation of conoid biogenesis and centrosome duplication, respectively. The third kinase, MAPK2, is specific to and conserved throughout the Alveolata, although its function is unknown. We used the auxin-inducible degron system to determine phenotypes associated with MAPK2 loss of function in *Toxoplasma*. We observed that parasites lacking MAPK2 failed to duplicate their centrosomes and therefore did not initiate daughter cell budding, which ultimately led to parasite death. MAPK2-deficient parasites initiated but did not complete DNA replication and arrested prior to mitosis. Surprisingly, the parasites continued to grow and replicate their Golgi apparatus, mitochondria, and apicoplasts. We found that the failure in centrosome duplication is distinct from the phenotype caused by the depletion of MAPKL1. As we did not observe MAPK2 localization at the centrosome at any point in the cell cycle, our data suggest that MAPK2 regulates a process at a distal site that is required for the completion of centrosome duplication and the initiation of parasite mitosis.

## Abstract (precis)

Toxoplasma gondii is a ubiquitous intracellular protozoan parasite that can cause severe and fatal disease in immunocompromised patients and the developing fetus. Rapid parasite replication is critical for establishing a productive infection. Here, we demonstrate that a *Toxoplasma* protein kinase called MAPK2 is conserved throughout the Alveolata and essential for parasite replication. We found that parasites lacking MAPK2 protein were defective in the initiation of daughter cell budding and were rendered inviable. Specifically, T. gondii MAPK2 (TgMAPK2) appears to be required for centrosome replication at the basal end of the nucleus, and its loss causes arrest early in parasite division. MAPK2 is unique to the Alveolata and not found in metazoa and likely is a critical component of an essential parasite-specific signaling network.

## INTRODUCTION

Cellular replication depends upon the faithful duplication and partition of genetic materials and cell organelles. Successful progression through the eukaryotic cell cycle is controlled by a network of well-conserved regulatory components that not only organize different stages but also adapt to extracellular signals (1). The obligate intracellular apicomplexan parasites have among the most diverse replicative paradigms among eukaryotes (2). These unusual cell cycles appear to be controlled by mechanisms both analogous to and divergent from those of their metazoan hosts (3–5). The Toxoplasma gondii asexual cycle replicates via “endodyogeny,” wherein two daughters grow within an intact mother (6). Efficient replication of these parasites is critical for the establishment of infection and also results in host tissue damage, thus contributing considerably to pathogenesis.

As the physical anchor for DNA segregation, the centrosome is central to the organization of cell division. Centrosome duplication is one of the earliest and most decisive events in the parasite cell cycle (3, 7–10). Previous work identified two distinct centrosomal cores, an outer core and an inner core, which orchestrate cytokinesis and nuclear division independently (3, 11). Daughter cell budding initiates prior to the completion of mitosis, and the parasite centrosome is a central hub that coordinates both mitosis and daughter budding. Budding begins at the end of S phase with daughter cytoskeletal components assembling near the duplicated centrosomes, which provide a scaffold for daughter cell assembly (8, 12, 13). Organelles are then partitioned between these elongating scaffolds (14). A growing number of regulatory components of the parasite cell cycle have been identified, including homologs of scaffolds (4, 15) and kinases (5, 8, 16, 17) that are broadly conserved in other organisms. However, the precise roles that these factors play in *Toxoplasma* are often distinct from those that they are known to play in model organisms; this is likely due to the parasite’s specialized cell cycle (6) and unusual centrosomal structure (3, 11). Thus, even the study of well-conserved proteins can yield surprising insight into the functional adaptations that evolved in the network that regulates the parasite cell cycle.

**FIG 1:**
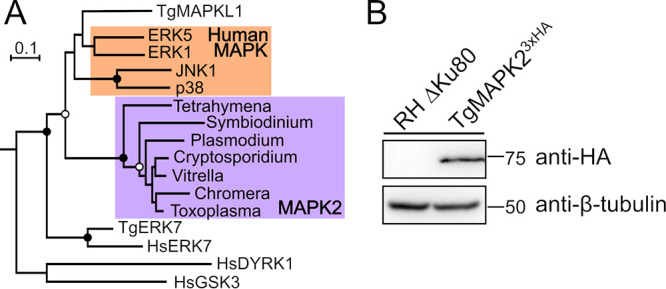
(A) Phylogenetic tree demonstrating that MAPK2 represents a distinct MAPK subfamily (highlighted in purple) that is conserved throughout the Alveolata. Human MAPKs ERK1, ERK5, Jun N-terminal protein kinase 1 (JNK1), and p38 are highlighted in orange. Human DYRK1, GSK3, and ERK7 and *Toxoplasma* MAPKL1 and ERK7 were used as outgroups. Black circles indicate bootstrap support of >95%, and open circles indicate bootstrap support of >80%. (B) Western blot of TgMAPK2^3×HA^ and parental lysates. β-Tubulin (anti-β-tubulin) was used as a loading control.

For example, throughout eukaryotes, members of the mitogen-activated protein kinase (MAPK) family are essential regulators of cell proliferation and differentiation (18–21). The *Toxoplasma* genome encodes three MAPKs: extracellular signal-regulated kinase 7 (ERK7), MAPKL1, and MAPK2 (Fig. 1A). ERK7 is conserved throughout the eukaryota, and we have recently shown that T. gondii ERK7 (TgERK7) is essential for conoid biogenesis (22). TgMAPKL1 is found only in coccidian parasites and plays a role in preventing centrosome overduplication in order to ensure proper binary division through endodyogeny (3, 23). We have identified the cellular function of the third kinase, MAPK2, which is specific to and conserved throughout the Alveolata. To uncover the function of MAPK2 in *Toxoplasma*, we applied the auxin-inducible degron (AID) system to inducibly degrade the protein. We found that parasites lacking TgMAPK2 arrested early in the cell cycle, which eventually led to parasite death. While these parasites failed to duplicate their centrosomes and never initiated daughter cell budding, they continued to grow and replicate their Golgi apparatus, mitochondria, and apicoplasts. Our data implicate TgMAPK2 as an essential regulator of an early checkpoint that is required to couple *Toxoplasma* cell growth with the completion of the cell cycle.

## RESULTS

### MAPK2 localizes to cytoplasmic puncta in *Toxoplasma*.

The *Toxoplasma* genome encodes three MAPKs. Two of these kinases have been characterized: TgMAPKL1 (TGME49_312570) prevents centrosome overduplication (3), and TgERK7 (TGME49_233010) is required for conoid biogenesis (22, 24). The gene TGME49_207820 encodes the *Toxoplasma* ortholog of MAPK2, a MAPK that is specific to and conserved in all alveolates, suggesting a specialized function (Fig. 1A). We first sought to determine the subcellular localization of TgMAPK2. To this end, we engineered a parasite strain to express native TgMAPK2 with a C-terminal 3-hemagglutinin (3×HA) epitope tag (TgMAPK2^3×HA^) using a CRISPR-mediated double-homologous-recombination strategy. A Western blot of the TgMAPK2^3×HA^ parasite lysate stained with anti-HA antibody showed a single band of the expected mass (66 kDa) (Fig. 1B).

**FIG 2:**
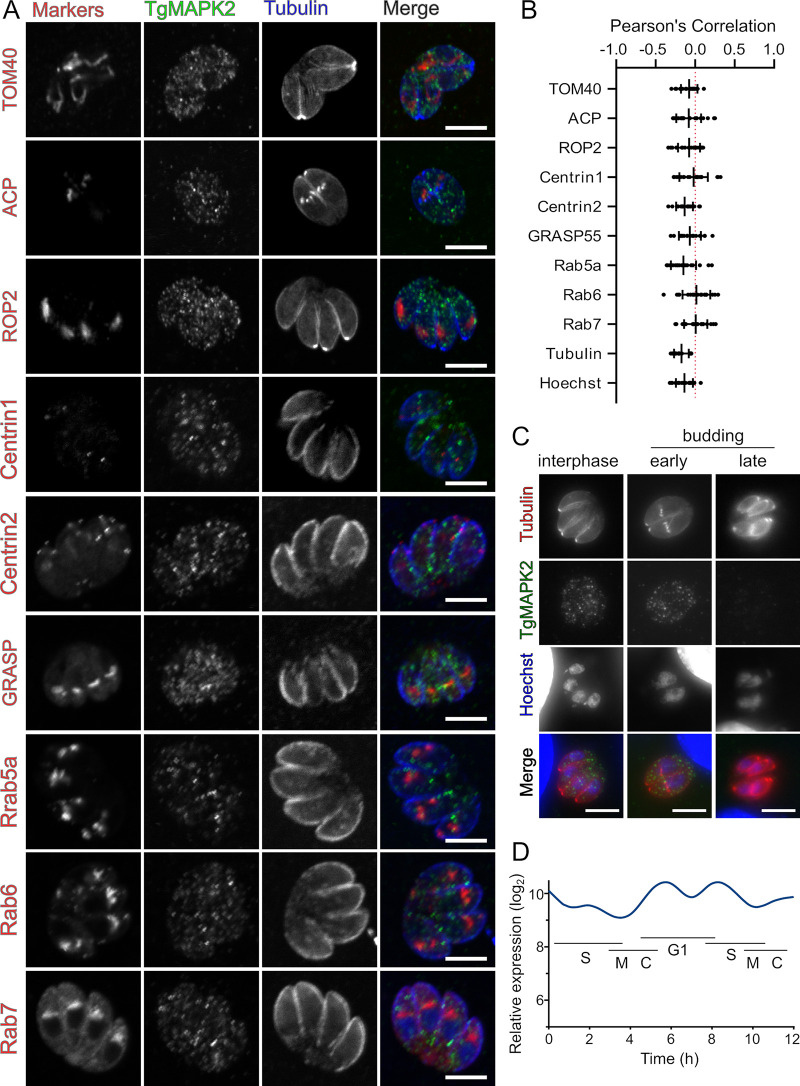
TgMAPK2 forms cytosolic puncta in interphase and early budding parasites. (A) Confocal 0.5-μm slices of TgMAPK2^3×HA^- and GFP–α-tubulin (blue)-expressing parasites were either stained or transiently transfected (Centrin and Rab FP fusions) with the indicated markers. (B) Pearson’s correlation coefficients for TgMAPK2^3×HA^ and the indicated markers from panel A (*n* = ∼20 cells). (C) TgMAPK2^3×HA^ intensity diminishes late in the cell cycle. (D) Transcript levels of MAPK2 peak during G_1_/early S (data from reference 26, retrieved from ToxoDBv43). Bars = 5 μm.

Our initial immunofluorescence analysis (IFA) revealed that TgMAPK2 appears as puncta dispersed throughout the parasite cytosol (Fig. 2A). We therefore costained parasites with anti-Tgβ-tubulin and several other well-characterized markers for parasite organelles. We observed no colocalization of TgMAPK2^3×HA^ puncta with any organellar markers that we tested (Fig. 2A and B), including β-tubulin, Hoechst stain, mitochondria (anti-TOM40), apicoplasts (anti-ACP), rhoptries (anti-ROP2), or centrosomes/conoids (Centrin1 and Centrin2). We reasoned that the TgMAPK2 puncta may represent intracellular vesicles and went on to test its colocalization with markers for intracellular trafficking, including the Golgi apparatus (GRASP55) and the endolysosomal trafficking system (*Toxoplasma* Rab proteins) (25). While many of these markers also appear punctate, confocal imaging revealed that TgMAPK2^3×HA^ does not localize to structures stained by any of GRASP55, Rab5a, Rab6, or Rab7 (Fig. 2A and B). TgMAPK2 therefore appears to mark a structure that is distinct from the well-characterized organelles and trafficking machinery in *Toxoplasma*. Moreover, we observed that the TgMAPK2 signal dropped to nearly undetectable levels late in the parasite cell cycle (Fig. 2C). While this loss of signal intensity may be due to the dissolution of the TgMAPK2 puncta, it is consistent with the variation of TgMAPK2 transcript levels throughout the parasite cell cycle, with a minimum during mitosis (Fig. 2D) (26).

We next used subcellular fractionation to attempt to determine whether TgMAPK2 behaved as a soluble protein or had an appreciable membrane or cytoskeletal interaction. TgMAPK2^3×HA^ parasites were lysed by freeze-thawing and ultracentrifuged to separate the soluble (high-speed supernatant [HSS]) and insoluble (high-speed pellet [HSP]) fractions. The HSP fraction was resuspended in either phosphate-buffered saline (PBS), 0.1 M Na_2_CO_3_ (pH 11.5) (high pH), 1 M NaCl (high salt), or 1% Triton X-100 (TX100) and incubated at 4°C for 30 min, followed again by ultracentrifugation. We found that while the vast majority of the TgMAPK2 protein was found in the first HSS fraction, a small portion of the protein was in the HSP fraction and was soluble only in detergent (see Fig. S1 in the supplemental material). Thus, TgMAPK2 behaves as a soluble protein, although a small portion of the protein (<5%) may be integrally associated with a cellular membrane.

10.1128/mBio.02517-20.1 FIG S1 Subcellular fractionation demonstrates that TgMAPK2^3×HA^ behaves as a soluble protein. TgMAPK2^3×HA^ parasites were lysed by freeze-thawing, and the lysate was ultracentrifuged at 120,000 × *g* for 2 h to separate the high-speed supernatant (1st HSS) and the high-speed pellet (1st pellet). The pellet was then extracted with either PBS, 0.1 M Na_2_CO_3_ (pH 11.5), 1 M NaCl, or 1% Triton X-100 (TX100) at 4°C for 30 min; resedimented by ultracentrifugation, as described above; separated by SDS-PAGE; and probed with anti-HA for Western blotting. Note that all lanes represent equivalent relative volumes. Download FIG S1, PDF file, 0.2 MB. Copyright © 2020 Hu et al. 2020 Hu et al. This content is distributed under the terms of the Creative Commons Attribution 4.0 International license.

### TgMAPK2 is essential for the completion of the parasite lytic cycle.

**FIG 3:**
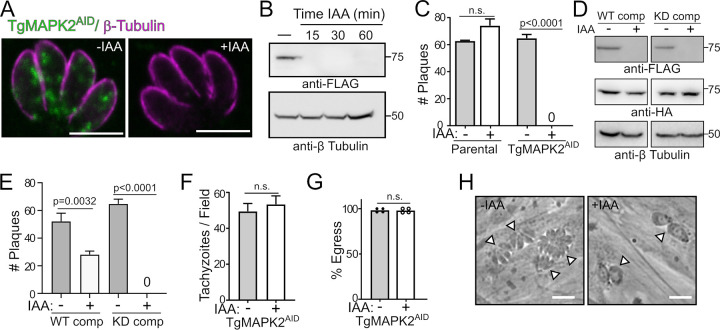
TgMAPK2 is essential for parasite proliferation. (A) Confocal 0.5-μm slices of TgMAPK2^AID^ parasites using anti-FLAG (green) and anti-Tgβ-tubulin (magenta) in the presence and absence of IAA. Bars = 5 μm. (B) Western blot of TgMAPK2^AID^ protein levels at increasing growth times in 500 μM IAA. Anti-Tgβ-tubulin was used as a loading control. (C) Quantification of data from triplicate plaque assays comparing parental and TgMAPK2^AID^ parasites grown in the presence and absence of IAA. (D) Protein levels of WT/KD-complemented TgMAPK2^3×HA^ parasites are not affected by IAA and do not affect the degradation of TgMAPK2^AID^. Anti-Tgβ-tubulin was used as a loading control. (E) Quantification of data from triplicate plaque assays comparing wild-type- or kinase-dead-complemented parasites grown in the presence and absence of IAA. (F and G) Invasion (F) and egress (G) of TgMAPK2 parasites grown without and, for the last 2 h, with IAA. (H) Phase-contrast micrograph comparing 20-h growth of TgMAPK^AID^ without and with IAA treatment. Arrowheads indicate individual vacuoles. Bars = 10 μm. *P* values are from two-tailed unpaired Student’s *t* test. n.s., not significant.

We were unable to obtain MAPK2 knockouts using either homologous recombination or CRISPR-mediated strategies, so we applied the AID system. The AID system allows the conditional degradation of target proteins upon the addition of a small molecule, auxin (indole-3-acetic acid [IAA]), and was recently adapted to *Toxoplasma* (27). We engineered a parasite strain in which the MAPK2 protein was expressed in frame with an AID and a 3×FLAG tag at the C terminus in the background of RHΔ*ku80* expressing the rice TIR1 auxin response protein (TgMAPK2^AID^). TgMAPK2 localization appeared unaffected by the addition of the AID tag, and TgMAPK2^AID^ was degraded upon the addition of 500 μM auxin (IAA) (Fig. 3A). TgMAPK2 protein was undetectable by Western blotting after 15 min of IAA treatment (Fig. 3B). We refer to parasites in which TgMAPK2 has been inducibly degraded as TgMAPK2^AID/IAA^. While TgMAPK2^AID^ parasites produced normal plaque numbers, TgMAPK2^AID/IAA^ parasites produced no plaques (Fig. 3C). To confirm that this phenotype is due to the absence of TgMAPK2 protein and to determine whether TgMAPK2 kinase activity is required for its function, we expressed an extra, nondegradable copy of wild-type (WT) or kinase-dead (KD) (D271A) kinase in the background of TgMAPK2^AID^ parasites (Fig. 3D). While the WT-complemented TgMAPK2^AID^ parasites were able to form plaques even in the presence of IAA, the KD-complemented parasites were not (Fig. 3E). TgMAPK2 kinase activity therefore appears required for its function. We note that WT-complemented parasites did not entirely rescue plaque formation. This is likely due to two factors: (i) we were unable to obtain stable clones of ectopically expressed MAPK2 driven by its native promoter and instead used the dihydrofolate reductase (DHFR) promoter, and (ii) ectopic MAPK2 expression appeared mosaic in a clonal population; 50 to 70% of parasites expressed the complemented copy at any given time, consistent with our rescue data. Taken together, the above-described data clearly demonstrate that TgMAPK2 is essential for the lytic cycle, which is consistent with our inability to genetically disrupt the TgMAPK2 gene.

Plaque assays report on the entire lytic cycle, comprising multiple rounds of invasion, replication, and egress. The degradation of TgMAPK2 did not significantly affect parasite invasion or egress from host cells (Fig. 3F and G). TgMAPK2^AID/IAA^ parasites did not, however, replicate normally. Instead, we observed that many TgMAPK2^AID/IAA^ parasites were able to undergo only a single round of replication following treatment with 500 μM IAA for 20 h, after which they arrested and showed an aberrant morphology by phase-contrast microscopy (Fig. 3H). While TgMAPK2^AID^ parasites replicated normally, more than 80% of the TgMAPK2^AID/IAA^ vacuoles contained two enlarged, morphologically aberrant parasites. The remaining TgMAPK2^AID/IAA^ vacuoles contained a single oversized parasite (Fig. 3H). These data led us to hypothesize that TgMAPK2 plays a crucial role at an early stage of parasite division.

### TgMAPK2 knockdown arrests parasites prior to initiation of daughter cell budding.

During acute infection, the *Toxoplasma* tachyzoite replicates by endodyogeny, in which two daughter cells are assembled within the mother (6). This mechanism of division requires that organellar biogenesis be tightly coupled to the cell cycle. The parasite cellular ultrastructure thus changes drastically as the cell cycle progresses, which provides us with a number of markers that can easily distinguish cell cycle stages via fluorescence microscopy and electron microscopy.

**FIG 4:**
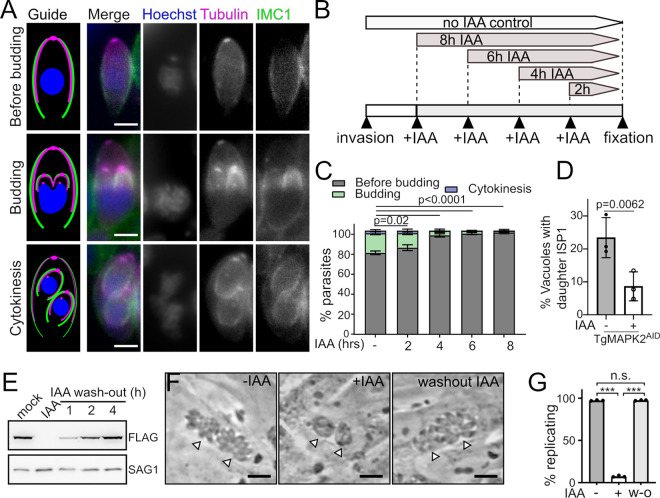
Loss of TgMAPK2 leads to a defect in daughter cell budding. TgMAPK2^AID^ parasites stably expressing mTFP1–α-tubulin (magenta) and TgIMC1-mVenus (green) were stained with Hoechst 33342 (blue) to distinguish the three indicated categories in the cell cycle. Bars = 2 μm. (B) Experimental flow of the addition of IAA in increasing 2-h increments. (C) Quantification of parasites in each category with increasing time of growth in IAA. Shown are means ± SD from 3 biological replicates; 200 to 300 parasites were counted under each condition. (D) Quantification of parasites with daughter cell budding rings (ISP1 in daughter buds) grown in the presence or absence of IAA for 8 h. Means ± SD from 3 biological replicates are shown; 200 to 300 parasites were counted under each condition. *P* values are from two-tailed Student’s *t* test. (E) Western blot demonstrating that MAPK2^AID^ protein levels are restored 2 to 4 h after IAA washout. (F) Phase-contrast micrograph comparing the growth of TgMAPK2^AID^ parasites over 18 h in IAA or after 2 h in IAA with an additional 16 h after washout. Arrowheads indicate individual vacuoles. Bars = 10 μm. (G) Quantification of the percentage of vacuoles that appeared to be replicating normally (≥4 parasites/vacuole) from 3 independent replicates. *P* values are from one-way analysis of variance (ANOVA) with Dunnett’s test (***, *P* < 0.0001). w-o, without.

In a normal culture, the *Toxoplasma* cell cycle is synchronized within individual vacuoles (28) but asynchronous among vacuoles in a population, even among parasites that have invaded at the same time. Because parasites divide asynchronously, at any given time, the parasites in a population occupy all stages of the cell cycle. We therefore reasoned that we could identify the point of arrest upon TgMAPK2 degradation by examining arrested parasites and determining which structures are lost during increasing times of growth in the presence of IAA. Here, we used a set of fluorescent markers to classify the parasite cell cycle into three broad categories: (i) “before budding,” (ii) “budding,” and (iii) “cytokinesis” (Fig. 4A). To distinguish these categories, we chose as markers (i) *Toxoplasma* inner membrane complex 1 (IMC1), which stains the mother outline and growing daughter scaffolds; (ii) β-tubulin, which labels the subpellicular microtubules and conoids; and (iii) Hoechst stain, which labels nuclear and plastid DNA. We engineered the TgMAPK2^AID^ strain to coexpress IMC1-mVenus and monomeric teal fluorescent protein 1 (mTFP1)–α-tubulin. TgMAPK2^AID^ parasites were allowed to invade a fresh monolayer of fibroblasts for 2 h, after which uninvaded parasites were washed off. Media were changed to media with IAA (+IAA media) after increasing 2-h increments, and all parasites were grown for a total of 10 h (Fig. 4A and B). Parasites were then fixed and prepared for imaging.

We quantified the number of parasites in each rough stage of the cell cycle for increasing incubation times in +IAA medium (Fig. 4C). We observed that parasites grown without IAA had 79% ± 2% parasites before budding, 19% ± 2% budding parasites, and 3% ± 1% parasites undergoing cytokinesis. As the time in IAA increased, we observed a marked decrease in the number of parasites that were budding and undergoing cytokinesis. After 6 to 8 h of IAA treatment, 98% ± 2% of the parasites were in a nonbudding state. Taken together, these data demonstrate that the arrest that we observe due to the degradation of TgMAPK2 occurs prior to the initiation of daughter cell budding, after which TgMAPK2 does not appear essential for division. To verify the defect in the initiation of daughter cell budding in the absence of TgMAPK2, we tested IMC subcompartment protein 1 (ISP1) labeling of parasites, which is an early marker for daughter bud formation (29). Consistent with the quantification of IMC1 staining (Fig. 4C), ISP1 staining demonstrated that daughter cell budding rings appeared in 23% ± 6% of parasites without IAA, whereas only 8% ± 4% of parasites had daughter budding rings after 8 h of IAA treatment (Fig. 4D).

A regulatory arrest in the cell cycle should be reversible by restoration of the protein. Induced degradation of an AID-tagged protein can be reversed by washing out IAA (Fig. 4E) (30). We therefore asked whether the block that we had observed in parasite division due to TgMAPK2 degradation was reversible. TgMAPK2^AID^ parasites were allowed to invade host fibroblasts and then grown for 8 h in the presence or absence of IAA. Either parasites were then allowed to continue to grow in IAA for an additional 18 h, or the medium was changed to medium without IAA (−IAA medium) before continued growth. Parasites were then fixed and imaged by phase-contrast microscopy. As expected, TgMAPK2^AID^ parasites grown continuously in IAA were arrested. Parasites that had been grown for only 8 h in IAA before washout, however, were indistinguishable from those that had been grown without IAA (Fig. 4F and G). Thus, the block in *Toxoplasma* division caused by TgMAPK2 degradation likely represents a checkpoint arrest.

### TgMAPK2 degradation impairs centrosome duplication.

**FIG 5:**
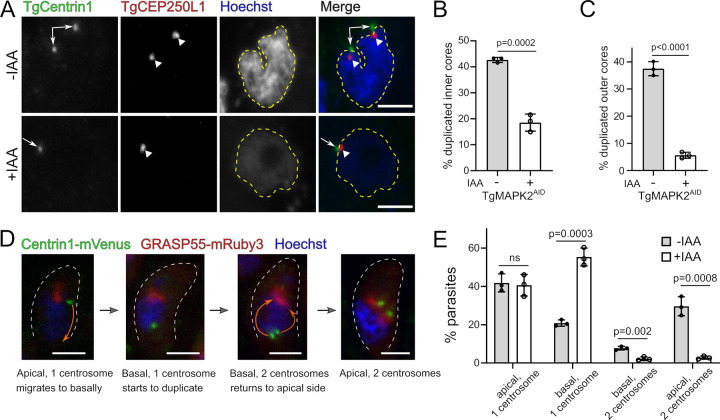
TgMAPK2 degradation impairs centrosome duplication after basal migration. (A) Z-projections of confocal stacks of TgMAPK2^AID^ parasites stably expressing mVenus-TgCentrin1 (green) and TgCEP250L1-3×HA (red) grown for 8 h in the presence (+) or absence (−) of 500 μM IAA and costained with Hoechst stain (blue). Arrows, TgCentrin1; large arrowheads, TgCEP250L1. Nuclei are outlined in yellow. (B) Quantification of parasites with duplicated centrosomal inner cores (TgCEP250L1) (B) and outer cores (TgCentrin1) (C) grown in the presence or absence of IAA for 8 h. Means ± SD from 3 biological replicates are shown; 200 to 500 parasites were counted under each condition. (D) Maximum-intensity Z-projections of confocal stacks of TgMAPK2^AID^ parasites stably expressing mVenus-TgCentrin1 (green) and GRASP55-mRuby3 (red) during the cell cycle. Representative images summarizing centrosome migration/duplication are shown. (E) Quantification of parasites in each category shown in panel D grown in the presence or absence of IAA for 8 h. Means ± SD from 3 biological replicates are shown; 300 to 500 parasites were counted under each condition. *P* values are from two-tailed Student’s *t* test. Bars = 2 μm.

An early, crucial event prior to *Toxoplasma* daughter cell budding is the duplication of the centrosome (8). Centriole duplication during the G_1_/S boundary of the cell cycle marks entry into the S phase and provides a spatial direction for the assembly of the daughter buds (31). Previous work identified an unusual bipartite structure of the centrosome in *Toxoplasma*, which includes two cores: the outer core, distal from the nucleus, and the inner core, proximal to the nucleus (3). These cores have distinct protein compositions, by which they can be easily distinguished (3). We therefore checked whether the duplication of either centrosomal core was compromised in TgMAPK2^AID/IAA^ parasites. We engineered the TgMAPK2^AID^ strain to stably express an mVenus fusion to the centrosomal outer core protein TgCentrin1 and a 3×HA fusion to the centrosomal inner core protein TgCEP250L1. Freshly invaded parasites were grown in the presence or absence of IAA for 8 h. Anti-HA was used to stain TgCEP250L1, and Hoechst 33342 was included to track the nuclear DNA (Fig. 5A). We quantified the percentage of parasites with two clearly separated centrosomes based on both inner core marker TgCEP250L1 and outer core marker mVenus-TgCentrin1 signals. As expected, ∼40% of TgMAPK2^AID^ parasites grown in the absence of IAA had successfully duplicated both their centrosomal inner cores and outer cores (Fig. 5B and C), consistent with previous reports (5, 8). In contrast, the depletion of MAPK2 profoundly suppressed the duplication of both cores, although the phenotype was less severe for the inner core than for the outer core (Fig. 5B and C).

During late G_1_ phase, the centrosome migrates to the basal end of the nucleus, where it duplicates and then separates. Subsequently, duplicated centrosomes traffic back to the apical end of the nucleus, where they reassociate with the Golgi apparatus (32). To check whether the nonduplicated centrosome migrates to the basal end, we engineered the TgMAPK2^AID^ strain to express an mVenus fusion to the centrosome marker TgCentrin1 and an mRuby3 fusion to the Golgi marker GRASP55. Freshly invaded parasites were grown in the presence or absence of IAA for 8 h, and Hoechst 33342 was included to track the nuclear DNA. We summarize the centrosome migration/duplication cycle into four steps, (i) “apical, 1 centrosome”; (ii) “basal, 1 centrosome”; (iii) “basal, 2 centrosomes”; and (iv) “apical, 2 centrosomes,” as shown in Fig. 5D. We quantified the number of parasites at each step in the presence or absence of IAA. As expected, we found that treatment with IAA drastically reduced the number of duplicated centrosomes (Fig. 5E). Intriguingly, IAA treatment resulted in an ∼2.6-fold increase (55.3% ± 5% versus 21% ± 2%) of parasites in which the centrosome had migrated to the basal end of the parasite but failed to duplicate. Therefore, we conclude that TgMAPK2 depletion does not affect centrosome migration to the basal end of the nucleus, while it compromises the duplication of the centrosome.

### Knockdown of TgMAPK2 results in a defect in DNA replication that impairs entry into mitosis.

**FIG 6:**
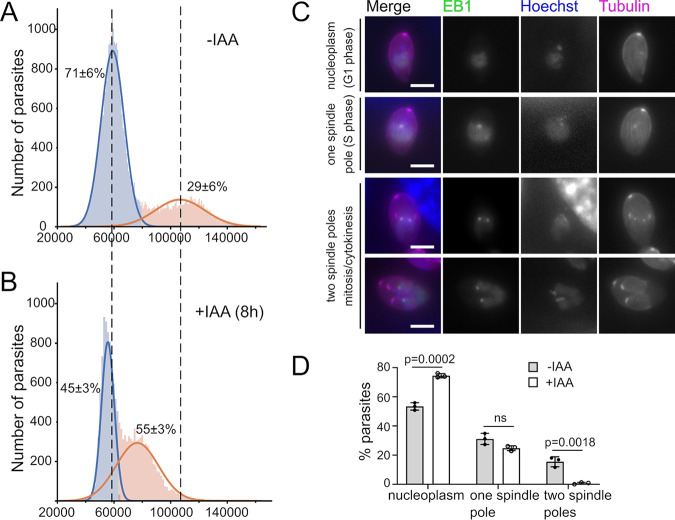
TgMAPK2 degradation results in a DNA replication defect and impairs mitosis. (A and B) Histograms showing DNA contents measured by FACS analysis. TgMAPK2^AID^ parasites growing in the presence or absence of IAA for the last 8 h were labeled with 1 μg/ml DAPI, and 50,000 events were collected from each of the three replicates. Gaussian mixtures were estimated for individual FACS runs, and the area under the Gaussian curves was measured. (C) TgMAPK2^AID^ parasites stably expressing TgEB1-mRuby3 (green) and mTFP1–α-tubulin (magenta) were imaged, together with Hoechst staining. Representative images for each stage are shown. Bars = 2 μm. (D) Quantification of parasites in each stage shown in panel C grown in the presence or absence of IAA for 8 h. Means ± SD from 3 biological replicates are shown; 80 to 200 parasites were counted under each condition. *P* values are from unpaired two-tailed Student’s *t* test.

DNA replication and nuclear division are crucial for each daughter cell to inherit one copy of the cell’s genetic material. We observed substantially weaker nuclear Hoechst staining in TgMAPK2^AID/IAA^ than in control parasites when imaged using the same microscope settings (Fig. 5A). As we observed this phenotype even after relatively short, 8-h growth in IAA, the low-Hoechst-intensity phenotype occurs early after the loss of TgMAPK2 and is not a secondary effect or artifact of prolonged growth in IAA. This phenotype suggests that DNA replication and karyokinesis may also be inhibited in TgMAPK2^AID/IAA^ parasites. To test this hypothesis, we stained the parasite DNA with 4′,6-diamidino-2-phenylindole (DAPI) and performed a fluorescence-activated cell sorter (FACS)-based analysis to compare the DNA content in TgMAPK2^AID^ to that in parasites grown for 8 h in IAA. A total of 71% ± 6% of control parasites possessed 1N DNA content, while 29% ± 6% of parasites possessed ∼1.8N to ∼2N DNA content (Fig. 6A). In TgMAPK2^AID/IAA^ parasites, however, the majority (55% ± 3%) of parasites possess >1N DNA content, although the peak is left shifted (Fig. 6B), indicating that TgMAPK2^AID/IAA^ parasites can initiate, but not complete, DNA replication. Thus, TgMAPK2^AID/IAA^ parasites are unable to progress successfully through S phase.

The coordination of spindle assembly and the centrosome cycle are critical for mitosis progression. During the *Toxoplasma* cell cycle, the formation of spindle poles is coincident with the completion of DNA replication and nucleus lobulation (14, 33). In order to determine whether mitosis initiates in TgMAPK2^AID/IAA^ parasites, we used microtubule end-binding protein 1 (EB1) as a marker. EB1 distributes in the nucleoplasm during interphase but transitions to the spindle poles when mitosis starts (33). The TgMAPK2^AID^ strain was engineered to stably express TgEB1-mRuby3. Freshly invaded parasites were grown in the presence or absence of IAA for 8 h, and Hoechst 33342 and β-tubulin were then included to track the nuclear DNA and mark the parasite boundary. We classified the localization of TgEB1 into (i) “nucleoplasm” (interphase), (ii) “one spindle pole” (S phase), and (iii) “two spindle poles” (mitosis/cytokinesis) (Fig. 6C). Next, we counted the parasite numbers in each class based on the TgEB1-mRuby3 signal. We observed that without IAA treatment, 53% ± 3% of TgMAPK2^AID^ parasites presented a nucleoplasmic TgEB1 localization, compared to 75% ± 1% of TgMAPK2^AID/IAA^ parasites. A total of 31% ± 4% of TgMAPK2^AID^ parasites and 25% ± 2% of TgMAPK2^AID/IAA^ parasites displayed one spindle pole, indicating successful entry into S phase. However, while 16% ± 3% of TgMAPK2^AID^ parasites showed two spindle poles of TgEB1 localization, essentially none of the TgMAPK2^AID/IAA^ parasites did (Fig. 6D). Taken together, our results indicate that TgMAPK2^AID/IAA^ parasites are able to enter S phase, where they arrest, unable to proceed into mitosis.

### Degradation of TgMAPK2 does not block replication of the mitochondrion, apicoplast, or Golgi apparatus.

**FIG 7:**
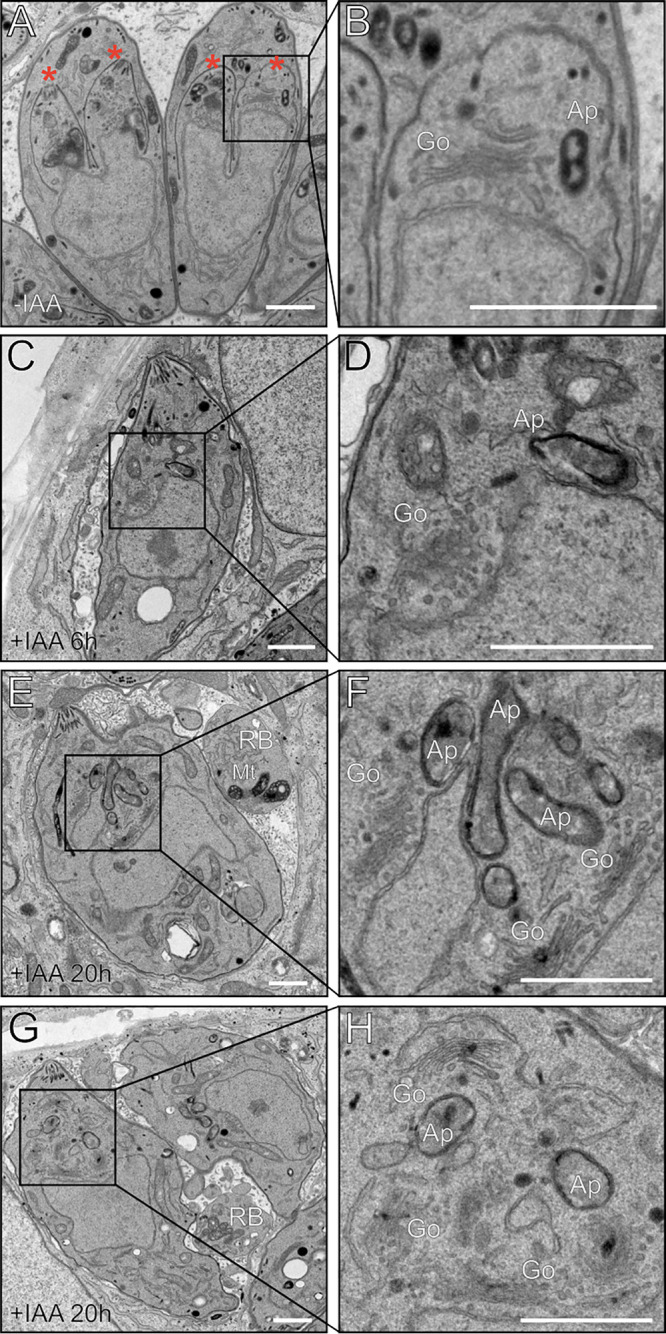
Prolonged incubation in IAA results in multiple defects in the TgMAPK2^AID^ parasite ultrastructure. Shown are transmission electron micrographs of intracellular TgMAPK2^AID^ parasites in the absence of IAA (A and B), with 6 h of growth in IAA (C and D), or with 20 h of growth in IAA (E to H). *, daughter buds; Go, Golgi apparatus; Ap, apicoplast; Mt, mitochondria; RB, residual bodies. Bars = 1 μm. Note that 100% of parasites treated for 20 h with IAA had overduplicated apicoplasts, and 35% ± 5% had overduplicated Golgi apparatus; neither phenotype was observed in untreated parasites.

To better understand the nature of the arrest upon TgMAPK2 depletion, we next sought to characterize the morphological changes of the parasite after this arrest. We used transmission electron microscopy (TEM) to compare the ultrastructures of TgMAPK2^AID^ parasites grown in the absence and the presence of IAA for a short time (6 h), which was sufficient to induce arrest. We observed well-formed daughter buds in ∼20% of the asynchronously dividing TgMAPK2^AID^ parasites (Fig. 5A), which is consistent with our IFA quantification (Fig. 4). While the TgMAPK2^AID/IAA^ parasites did not show obvious ultrastructural defects, daughter cell budding appeared completely blocked (Fig. 7C), again consistent with our IFA quantification. We next examined parasites that were incubated for extended periods in IAA. After 20 h in IAA, the architecture of TgMAPK2^AID/IAA^ parasites was misshapen, as evidenced by enlarged cells and the loss of the parasite’s distinctive shape (Fig. 7E to H). In addition, we observed an increase in the cellular loads of many organelles, including the Golgi apparatus and apicoplasts (Fig. 7E to H). Moreover, at these later time points, we observed vacuoles containing enlarged residual bodies filled with organellar debris, even in vacuoles containing only a single (albeit misshapen) parasite (Fig. 7E).

**FIG 8:**
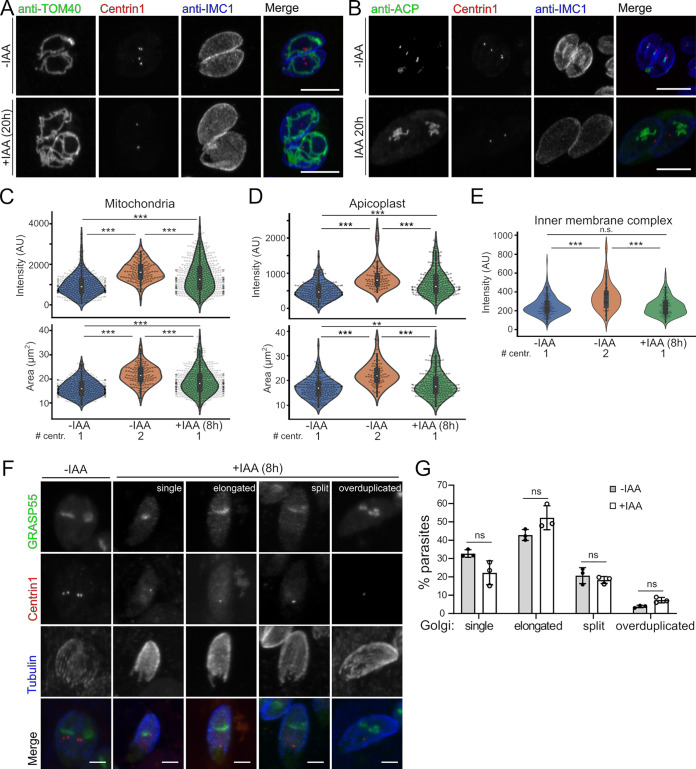
Loss of TgMAPK2 does not block organelle replication. (A and B) Z-projections of confocal stacks of TgMAPK2^AID^ parasites stably expressing mVenus-TgCentrin1 (red) grown for 20 h in the presence or absence of IAA. Parasites were fixed and stained with antibodies for IMC1 (blue), TOM40 (green), or ACP1 (green). Bars = 5 μm. (C to E) The total intensities of TOM40 (mitochondria), ACP (apicoplasts), and IMC1 as well as the areas of the same set of parasites were measured and quantified for each class. AU, arbitrary units. (F) Z-projections of confocal stacks of TgMAPK2^AID^ parasites stably expressing mVenus-TgCentrin1 (red) and TgGRASP55-mRuby3 (green) grown in the presence or absence of IAA for 8 h and costained with anti-Tgβ-tubulin. Headings in GRASP55 panels indicate Golgi morphological class descriptions. Representative images for each class presenting Golgi morphologies are shown. (G) Quantification of parasites in each class shown in panel F grown in the presence or absence of IAA for 8 h. Means ± SD from 3 biological replicates are shown; 200 to 300 parasites were counted under each condition. *P* values are from two-tailed Student’s *t* test.

In order to scrutinize organelle replication progress and centrosome duplication simultaneously in TgMAPK2^AID/IAA^ parasites, mVenus-Centrin1-expressing parasites were stained with antibodies recognizing either TOM40 (mitochondrion) or ACP1 (apicoplast). Individual parasites and their daughter buds were identified by IMC1 staining. Parasites were grown for 20 h in the presence or absence of IAA. Our microscopic analysis confirmed that TgMAPK2^AID^ parasites replicated normally and maintained normal counts of apicoplasts and mitochondria per parasite (Fig. 8A and B). Strikingly, in TgMAPK2^AID/IAA^ parasites, mitochondria and apicoplasts continued to grow even after arrest, although without centrosome duplication and new daughter cell buds, these organelles failed to separate and partition (Fig. 8A and B).

We next asked whether the increased apicoplast and mitochondrial loads that we observed after 20 h of growth in IAA were also observable at earlier time points. Using the same staining strategy as the one described above, parasites were grown in the presence or absence of IAA for 8 h before analysis. As >95% of TgMAPK2^AID/IAA^ parasites possessed a single centrosome at this time point in our data set, we focused on this majority population. We quantified the intensity of organellar staining as a proxy for organelle load in both control and IAA-treated parasites. As expected, control parasites with duplicated centrosomes had increased mitochondrial and apicoplast loads compared to cells with a single centrosome. TgMAPK2^AID/IAA^ parasites, however, displayed a nonnormal distribution of mitochondrial and apicoplast loads that was significantly different from those of both control populations (Fig. 8C and D). Using the IMC1 signal as a guide, we quantified the size of each parasite. As expected, control parasites that had initiated budding were consistently larger than single-centrosome parasites (Fig. 8C and D). TgMAPK2^AID/IAA^ parasites, however, had a complex distribution of sizes that was again distinct from those of both control populations.

All organisms’ cells match their organellar load to their need, which is quite often correlated with the cell’s size. We found that for all populations described above, cell size was correlated with apicoplast and mitochondrial loads (Fig. S2). To confirm that this observation was specific and not due to an artifact of image analysis, we reanalyzed the ACP1-stained data set to quantify IMC1 levels (Fig. 8E). Consistent with the idea that the IMC is created during cell division (34) rather than throughout growth, we found that control parasites with 2 centrosomes had higher levels of IMC1 than those with a single centrosome (Fig. 8E). Importantly, IMC1 levels in IAA-treated cells were indistinguishable from those in control parasites with a single centrosome (Fig. 8E), and IMC1 levels were constant across cell size in IAA-treated cells (Fig. S2).

10.1128/mBio.02517-20.2 FIG S2 Scatterplot illustrating the total-intensity trend of TOM40 (mitochondrion), ACP (apicoplast), and IMC1. −IAA parasites are binned by centrosome number [(1) or (2)], whereas all parasites included in the +IAA data set had only 1 centrosome. Trend lines represent linear regression for the +IAA (green) and combined −IAA (gray) data sets, and shaded bands represent 95% confidence intervals. Note that the −IAA data sets were combined for linear regression, as the one/two-centrosome bins tended to cluster in separate quadrants. Data indicate that mitochondrial and apicoplast loads, quantified by TOM40 and ACP1 intensities, respectively, were correlated with parasite size. IMC1 intensity, however, correlated more with centrosome numbers than parasite size and was essentially constant in TgMAPK2^AID/IAA^ parasites irrespective of their size (note that an *R*^2^ equal to 0 for linear regression indicates that a horizontal line is the best model for the data). Download FIG S2, PDF file, 0.4 MB. Copyright © 2020 Hu et al. 2020 Hu et al. This content is distributed under the terms of the Creative Commons Attribution 4.0 International license.

Centrosome duplication and Golgi division are two of the earliest events in the highly organized parasite cell cycle (14). The centrosomes are always associated with the Golgi apparatus except ∼1 h of migration to the basal end of the nucleus (14, 32). To examine the relationship between centrosome duplication and Golgi behaviors, we used TgMAPK2^AID^ parasites that stably express both mVenus-Centrin1 and the Golgi marker GRASP55-mRuby3. Parasites were grown with or without IAA for 8 h, and β-tubulin was visualized to mark the parasite boundary. We binned and quantified the Golgi morphologies as (i) “single,” (ii) “elongated,” (iii) “split,” and (iv) “overduplicated” based on the GRASP55-mRuby3 signal (Fig. 8F). Our analysis showed that the Golgi apparatus appeared to replicate normally in TgMAPK2^AID/IAA^ parasites, even without centrosome duplication (Fig. 8G).

Taken together, these data demonstrate that without TgMAPK2, cell and organellar growth continues unhindered and is therefore uncoupled from the parasite cell cycle.

### The two MAPKs MAPKL1 and MAPK2 are required at different checkpoints during the cell cycle.

**FIG 9:**
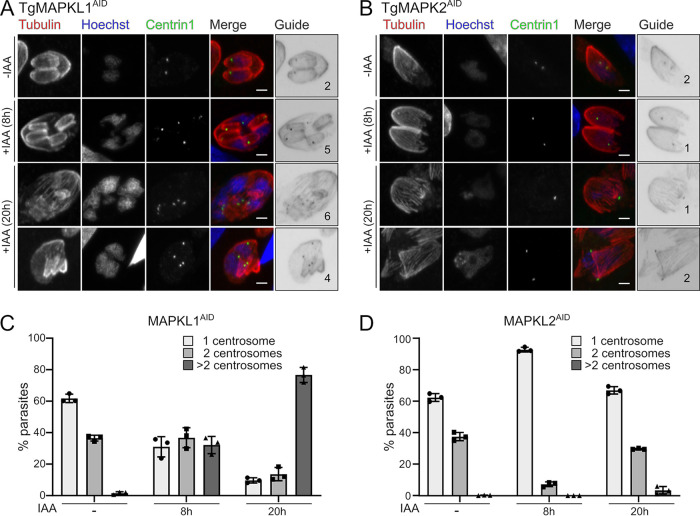
The centrosome duplication defect of TgMAPK2 is distinct from the phenotype caused by the depletion of TgMAPKL1. (A and B) TgMAPKL1^AID^ or TgMAPK2^AID^ parasites expressing TgCentrin1-mVenus (green) were treated with 500 μM IAA for 8 or 20 h and costained with anti-Tgβ-tubulin (red) and Hoechst stain (blue). Guide panels are merges of Centrin1 and β-tubulin signals, and numbers indicate the number of centrosomes. Bars = 2 μm. (C and D) Quantification of parasites with single, duplicated, or overduplicated centrosomes (TgCentrin1) grown in the presence or absence of IAA for 8 or 20 h. Means ± SD from 3 biological replicates are shown; 200 to 500 parasites were counted under each condition.

Our data show that TgMAPK2 is required for the replication of both centrosomal cores. Intriguingly, the loss of function of another member of the MAPK family, TgMAPKL1, also causes a defect in parasite centrosome replication (3). Notably, the previous study was performed using a temperature-sensitive mutant that necessitated studies at longer time scales (20 h at a nonpermissive temperature) and for which we observed secondary effects using the AID system with MAPK2. We therefore sought to compare the phenotypes caused by the depletion of these two MAPKs at both 8 h and 20 h. To this end, we generated a TgMAPKL1^AID^ strain using the same strategy as the one that we used for TgMAPK2. Freshly invaded TgMAPK2^AID^ or TgMAPKL1^AID^ parasites expressing mVenus-Centrin1 were grown in the presence of IAA for 8 or 20 h. Parasites were fixed and stained with anti-Tgβ-tubulin. We found that after 20 h with IAA, ∼30% of TgMAPK2^AID/IAA^ parasites possessed >1 centrosome, compared with ∼5% at 8 h of IAA treatment (Fig. 9C and D). Consistent with the previously reported data (3), and in contrast to the TgMAPK2^AID/IAA^ phenotype, we observed that 32.2% ± 5.4% of TgMAPKL1^AID/IAA^ parasites overduplicated their centrosomes (i.e., >2) after 8 h of IAA treatment, and 76.7% ± 4.8% of TgMAPKL1^AID/IAA^ parasites underwent centrosome overduplication after 20 h of IAA treatment (Fig. 9A and C). Thus, these two divergent MAPKs both regulate centrosome replication, although they have opposing phenotypes, and are thus likely to control different checkpoints in the *Toxoplasma* cell cycle.

## DISCUSSION

**FIG 10:**
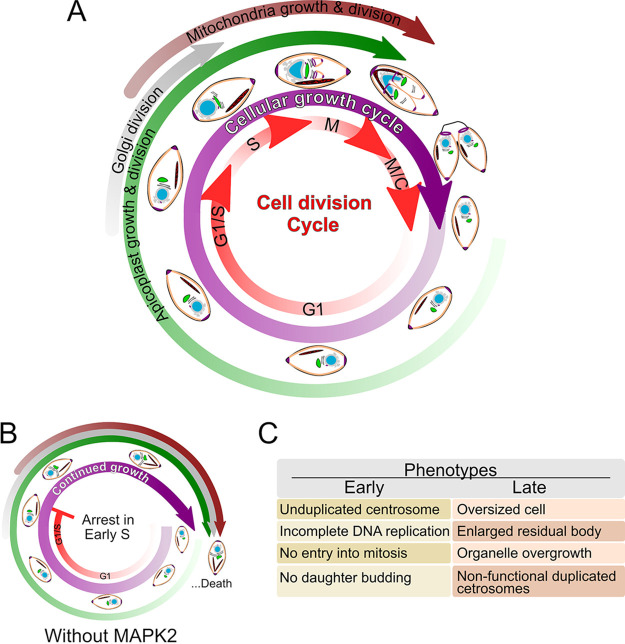
Loss of TgMAPK2 uncouples *Toxoplasma* organelle growth from cell division progression. (A) In normal parasites, cellular and organellar growth is synchronized with the division cycle. Cartoon representations indicate approximate parasite morphologies throughout the cell cycle. Approximate timings of organellar division are indicated by the colored arrows in the outer circles and are adapted from results described previously by Nishi et al. (14). (B) In MAPK2-depleted parasites, parasites appear to arrest in early S phase of the cell cycle but continue growing and increasing the organelle load. (C) Table summarizing early (8 h)- and late (18+ h)-appearing phenotypes observed upon MAPK2 depletion.

We have identified TgMAPK2 as essential for progression through an early checkpoint in the *Toxoplasma* cell cycle. Our data demonstrate that without TgMAPK2, parasites arrest before centrosome duplication, causing a block in the initiation of parasite budding and eventual parasite death. Intriguingly, organelles, including the Golgi apparatus, apicoplasts, and mitochondria, all continue to replicate, and the relative parasite size increases, even though budding does not occur (Fig. 10A to C). The process of endodyogeny has historically been described as a careful coordination of organellar biogenesis with parasite budding (6, 14, 35). Our data suggest that organelle biogenesis and cell growth are not directly coupled to the cell cycle through regulatory checkpoints but rather through simple timing. This idea is borne out by the ability to produce otherwise normal parasites in which the apicoplast does not replicate (36, 37). In addition, while the residual body is usually first observed after the first round of replication (38) and has recently been linked to interparasite trafficking within a vacuole (28), we observed enlarged residual bodies in vacuoles containing single, arrested parasites after long-term (20-h) TgMAPK2 degradation. Our data are therefore consistent with the role of the residual body in the response to cellular stress. Notably, treatment of parasites with pyrrolidine dithiocarbamate blocks *Toxoplasma* growth and leads to enlarged residual bodies, which was interpreted as a mechanism of cell size control (39).

While the depletion of TgMAPK2 results in reversible arrest just prior to centrosome duplication, after longer periods of growth without TgMAPK2, parasites often had multiple Centrin1-positive puncta (Fig. 9), which appear to represent partially formed or otherwise nonfunctional centrosomes. While these puncta were associated with microtubules, the tubulin structures were not well organized as in normal parasites (Fig. 9). Similarly, we did not observe these puncta associated with the parasite nuclei or organelles. This suggests a partial escape from arrest in which parasites lacking MAPK2 progress down an abnormal and lethal pathway. Similar scenarios have been well documented in cell cycle mutants in other organisms (40, 41).

A number of kinases with orthologs in model organisms have been identified as being critical for the *Toxoplasma* cell cycle. These include kinases in the cyclin-dependent kinase (CDK) (5, 16), Aurora (17, 42), and NIMA-related kinase (NEK) (8) families. Progression through G_1_/S is a critical control point throughout the eukaryota, and parasite kinases appear to perform functions similar to those of their metazoan orthologs in regulating this checkpoint. Notably, TgCRK1, an ortholog of metazoan CDK11, appears to regulate the transcriptional program that allows progression through the G_1_ checkpoint and centrosome duplication (5). In addition, TgNEK-1 is essential for centrosome separation (8). However, the ortholog of its mammalian substrate, CEP250, does not appear to be a substrate of TgNEK-1. In fact, CEP250 and TgNEK-1 show distinct phenotypes upon disruption (4). Thus, while proteins phylogenetically orthologous to those in well-studied models exist in apicomplexan parasites, they may not be functionally orthologous.

In addition to well-conserved kinases, there are a number of Apicomplexa-specific members of many families that typically control the eukaryotic cell cycle (5, 43, 44). In *Toxoplasma*, these include two specialized MAPKs. MAPKL1 has been found exclusively in coccidian organisms, which all replicate by endodyogeny during their asexual cycles. MAPK2 is conserved among all extant Alveolata for which genomes are available. While TgMAPKL1 localizes exclusively to the centrosome and prevents its overduplication (3), we have found that TgMAPK2 is required to complete a single round of centrosome duplication. We also found that TgMAPK2 never localizes to the centrosome or the nucleus. It thus seems likely that TgMAPK2 regulates centrosome duplication indirectly by controlling another process at a distal site that must be completed to progress through this checkpoint. The one unifying feature of all alveolate organisms is the membrane and cytoskeletal structure known as the inner membrane complex (IMC) in Apicomplexa and as “alveoli” in other organisms. The processes that regulate and drive IMC biogenesis are still a mystery. However, the IMC forms the scaffold for new daughter cells (6, 34), a process that is thought to be coordinated by the recently duplicated centrosome (8–10, 12, 34). Given its evolutionary history, it is intriguing to hypothesize that TgMAPK2 plays a role in the cross talk between these two processes.

## MATERIALS AND METHODS

### Sequence analysis and phylogeny.

Protein sequences for kinases were obtained from ToxoDBv43 and UniProt. The kinase domains were aligned using MAFFT (45), and the resulting alignments were used to estimate the maximum likelihood phylogenetic tree with bootstrap analysis (1,000 replicates) in IQ-tree v1.6.12 (46, 47), with the gamma-corrected empirical-frequency LG substitution model.

### PCR and plasmid generation.

All PCRs were conducted using Q5 DNA polymerase (New England BioLabs) and the primers listed in Table S1 in the supplemental material. Constructs were assembled using Gibson master mix (New England BioLabs).

10.1128/mBio.02517-20.3 TABLE S1 Primers used in this study. Download Table S1, XLSX file, 0.01 MB. Copyright © 2020 Hu et al. 2020 Hu et al. This content is distributed under the terms of the Creative Commons Attribution 4.0 International license.

### Parasite culture and transfection.

Human foreskin fibroblasts (HFFs) were grown in Dulbecco’s modified Eagle’s medium (DMEM) supplemented with 10% fetal bovine serum and 2 mM glutamine. Toxoplasma tachyzoites were maintained in confluent monolayers of HFFs. TgMAPK^3×HA^ and TgMAPK2^AID^ strains were generated by transfecting the RHΔ*ku80*Δ*hxgprt* strain (48) or the same strain expressing OsTIR1 driven by the gra1 promoter (22). Transfections included a CRISPR/Cas9 vector targeting the TgMAPK2 or TgMAPKL1 3′ end and a Q5 PCR product with 500-bp homology arms flanking the appropriate tag together with 10 μg of a Cas9 plasmid. TgMAPK2-complemented parasites were created by targeting 3×HA-tagged TgMAPK2 (WT or kinase dead) driven by the DHFR promoter, together with a hypoxanthine-xanthine-guanine phosphribosyl transferase (HXGPRT) cassette, to the empty Ku80 locus. mTFP1–α-tubulin-, green fluorescent protein (GFP)–α-tubulin-, TgIMC1-mVenus-, mVenus-TgCentrin1-, GRASP55-mRuby3-, or TgEB1-mRuby3-expressing parasites were created by amplifying the fluorescent protein (FP) marker expression cassette and an adjacent chloramphenicol (or HXGPRT) resistance cassette by PCR and targeting it to a site adjacent to the Ku80 locus by CRISPR/Cas9-mediated homologous recombination (Table S1) and selection with chloramphenicol (or mycophenolic acid and xanthine [MPA/Xan]). TgCEP250L1^3×HA^ parasites were generated by C-terminal single homologous recombination as described previously (3). The original pTub and pMIN vector was a kind gift of Ke Hu (University of Indiana). The original GRASP55-GFP is driven by the pTub promoter, and neon-Rab5a, emerald fluorescent protein (EmFP)-Rab6, neon-Rab7, emerald-Centrin1, and enhanced GFP (eGFP)-Centrin2 are all driven by the pMIN promoter, which was a kind gift of Aoife Heaslip (University of Connecticut).

### Western blotting.

Proteins were separated by SDS-PAGE and transferred to a polyvinylidene difluoride membrane. Membranes were blocked for 1 h in PBS plus 5% milk, followed by overnight incubation at 4°C with primary antibody in blocking solution. The next day, membranes were washed three times with Tris-buffered saline–Tween (TBST), followed by incubation at room temperature for 1 to 2 h with horseradish peroxidase (HRP)-conjugated secondary antibody (Sigma) in blocking buffer. After three washes with TBST, Western blots were imaged using ECL Plus reagent (Pierce) on a GE ImageQuant LAS4000 instrument. Antibodies used in this study include Rabbit (Rb) anti-Tgβ-tubulin (1:10,000 dilution), rat anti-HA (Sigma) (1:1,000 dilution), and mouse m2 anti-FLAG (Sigma) (1:1,000 dilution).

### Immunofluorescence and image analysis.

HFF cells were grown on coverslips in 24-well plates until confluent and were infected with parasites. The cells were rinsed twice with PBS and fixed with 4% paraformaldehyde (PFA)–4% sucrose in PBS at room temperature for 15 min. After two washes with PBS, cells were permeabilized with 0.1% Triton X-100 for 15 min and washed three times with PBS. After blocking in PBS plus 3% bovine serum albumin for 30 min, cells were incubated with primary antibody in blocking solution overnight at room temperature. Cells were then washed three times with PBS and incubated with Alexa Fluor-conjugated secondary antibodies (Molecular Probes) for 2 h. Cells were then washed three times with PBS and mounted with mounting medium containing DAPI (Vector Laboratories). For tyramide amplification of the TgMAPK2^AID-3×FLAG^ signal, the above-described protocol was altered as follows. Endogenous peroxidase activity was quenched by incubation of fixed coverslips with 100 mM sodium azide in PBS for 45 min at room temperature. Cells were blocked with 5% horse serum–0.5% Western Blocking Reagent Solution (Sigma-Aldrich) in TBST for 45 min. HRP-conjugated goat anti-mouse secondary antibody (Sigma) was used, and the tyramide fluorophore was allowed to react for 30 s before final washes. Cells were imaged on either a Nikon A1 confocal laser scanning microscope or a Nikon Ti2E wide-field microscope. Primary antibodies used in this study include rat anti-HA 3F10 (catalog number 11867423001; Roche), mouse m2 anti-FLAG (catalog number F1804; Sigma), rabbit anti-Tgβ-tubulin (1:10,000 dilution), rabbit anti-TOM40 (1:2,000 dilution), rabbit anti-ACP (1:2,000 dilution), rabbit anti-ROP2 (1:10,000 dilution), and mouse monoclonal antibody (MAb) 45.36 anti-TgIMC1 (1:2,000 dilution) (a generous gift from Gary Ward, The University of Vermont). Pearson’s coefficient was calculated for all the Z-stacks of the images of a minimum of 20 cells using Coloc 2 software in ImageJ for each of the markers in Fig. 2A and B. Quantitative image analysis in Fig. 8 and Fig. S2 was performed with ImageJ, as follows. All channels were background subtracted, and individual parasites were identified manually using the IMC1 signal. The total intensity of ACP1, IMC1, and TOM40 signals and the cell area were quantified and correlated on a per-cell basis.

### Membrane extraction analysis.

The freeze-thawed lysate from TgMAPK2^3×HA^ parasites was ultracentrifuged at 120,000 × *g* for 2 h to separate the soluble supernatant and pellet. The pellets were resuspended and extracted in either PBS, 0.1 M Na_2_CO_3_ (pH 11.5), 1 M NaCl, or PBS plus 1% Triton X-100 at 4°C for 30 min, followed by ultracentrifugation at 120,000 × *g*. Samples were analyzed by Western blotting as described above.

### Plaque assay.

Plaque assays were performed using 6-well plates containing HFFs infected with 200 parasites per well in the presence or absence of 500 μM IAA. After 7 days, the cells were fixed with methanol and stained with a crystal violet solution, and the resulting plaques were counted. All plaque assays were performed in biological triplicate.

### Transmission electron microscopy.

Cells were fixed on MatTek dishes with 2.5% (vol/vol) glutaraldehyde in 0.1 M sodium cacodylate buffer. After three rinses in 0.1 M sodium cacodylate buffer, the cells were postfixed with 1% osmium tetroxide and 0.8% K_3_[Fe(CN)_6_] in 0.1 M sodium cacodylate buffer for 1 h at room temperature. Cells were rinsed with water and *en bloc* stained with 2% aqueous uranyl acetate overnight. After three rinses with water, specimens were dehydrated with increasing concentrations of ethanol, infiltrated with Embed-812 resin, and polymerized in a 70°C oven overnight. Blocks were sectioned with a diamond knife (Diatome) on a Leica Ultracut UC7 ultramicrotome (Leica Microsystems) and collected onto copper grids poststained with 2% uranyl acetate in water and lead citrate. All TEM images were acquired on a Tecnai G2 Spirit transmission electron microscope (FEI) equipped with a LaB_6_ source at 120 kV. Images in Fig. 7B of sectioned cells are representative of 15 TgMAPK2^AID^, 40 TgMAPK2^AID/IAA^ (6-h IAA duration), and 40 TgMAPK2^AID/IAA^ (20-h IAA duration) vacuoles. The numbers of Golgi apparatus per parasite were counted manually from 35 images.

### Invasion and egress assays.

Invasion and egress assays were performed using mTFP1–α-tubulin-expressing TgMAPK2^AID^ parasites. Parasites were allowed to grow overnight without IAA. The next day, 2 h before the assay was initiated, the medium was switched to IAA (or mock) medium. Parasites were mechanically released from host cells, and 2 × 10^6^ parasites under each condition were added to confluent HFFs grown on coverslips in a 24-well plate, where they were allowed to invade at 37°C for 2 h in −/+IAA medium. These cells were then washed 10 times with PBS and fixed, and attached parasites were stained with anti-SAG1 without permeabilization. Assays were conducted in biological triplicates each of technical triplicates. The mTFP1–α-tubulin-positive but SAG1-negative parasites were regarded as internal (invaded) parasites. To measure egress, parasites were grown for 24 to 30 h in confluent HFFs on coverslips. Two hours before the assay was initiated, the medium was switched to IAA medium, as described above. Cells were washed with prewarmed Hanks’ balanced salt solution (HBSS) before the assay and then incubated with HBSS containing 3 μM calcium ionophore A23187 (Cayman Chemical) for 1 min at 37°C before fixation and imaging.

### DNA content analysis by FACS.

Parasite nuclear DNA content was determined by FACS analysis using 1 μg/ml DAPI. Intracellular parasites were washed with cold PBS twice, filtered with 5-μm filters, and fixed in ice-cold 80% (vol/vol) ethanol overnight. The parasites were pelleted at 300 × *g* and resuspended in 1.0 ml of a freshly made 1-μg/ml DAPI–Triton X-100 solution at room temperature for 30 min. Data were fit to Gaussian mixture models and plotted using the CytoFlow v1.0 python package (49).

### Statistical analysis and figure generation.

Statistical tests were conducted in GraphPad Prism v8.4. All data shown are means, and error bars indicate standard deviations (SD). Images were analyzed using the Fiji distribution of ImageJ (50). Data in Fig. 8 and Fig. S2 were plotted using the Seaborn python package, and statistical tests were performed in statsmodels. Figures were created and edited using Inkscape v0.92.
