# Supplementary material for: Loss of the Conserved Alveolate Kinase MAPK2 Decouples Toxoplasma Cell Growth from Cell Division
Source: mBio. 2020 Nov 10;11(6):e02517-20. doi: 10.1128/mBio.02517-20 (PMC7667025; doi:10.1128/mBio.02517-20)
Supplement: FIG S1 [file mBio.02517-20-sf001.pdf]

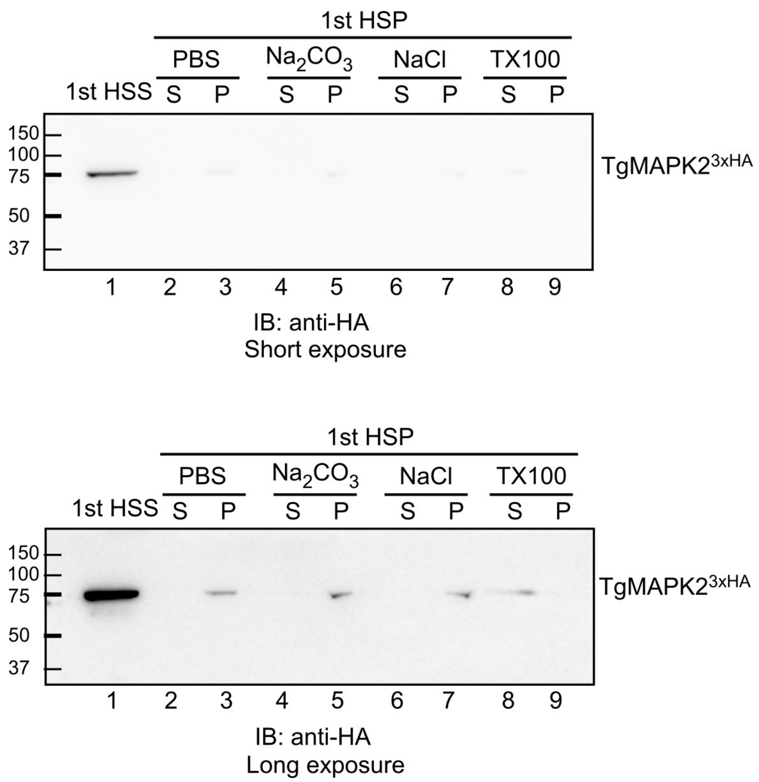

Supplemental Figure S1. Subcellular fractionation demonstrates that TgMAPK2^{3xHA} behaves as a soluble protein. TgMAPK2^{3xHA} parasites were lysed by freeze-thaw and the lysate was ultracentrifuged at 120,000 g for 2 h to separate the high speed supernatant (1st HSS) and high speed pellet (1st pellet). The pellet was then extracted with either PBS; 0.1 M Na_2CO_3, pH 11.5; 1 M NaCl or 1% TritonX-100 (TX100) at 4°C for 30 minutes, resedimented by ultracentrifugation, as above, separated by SDS-PAGE and probed with anti-HA for western blot. Note all lanes represent equivalent relative volumes.
