# Supplementary material for: Loss of the Conserved Alveolate Kinase MAPK2 Decouples Toxoplasma Cell Growth from Cell Division
Source: mBio. 2020 Nov 10;11(6):e02517-20. doi: 10.1128/mBio.02517-20 (PMC7667025; doi:10.1128/mBio.02517-20)
Supplement: FIG S2 [file mBio.02517-20-sf002.pdf]

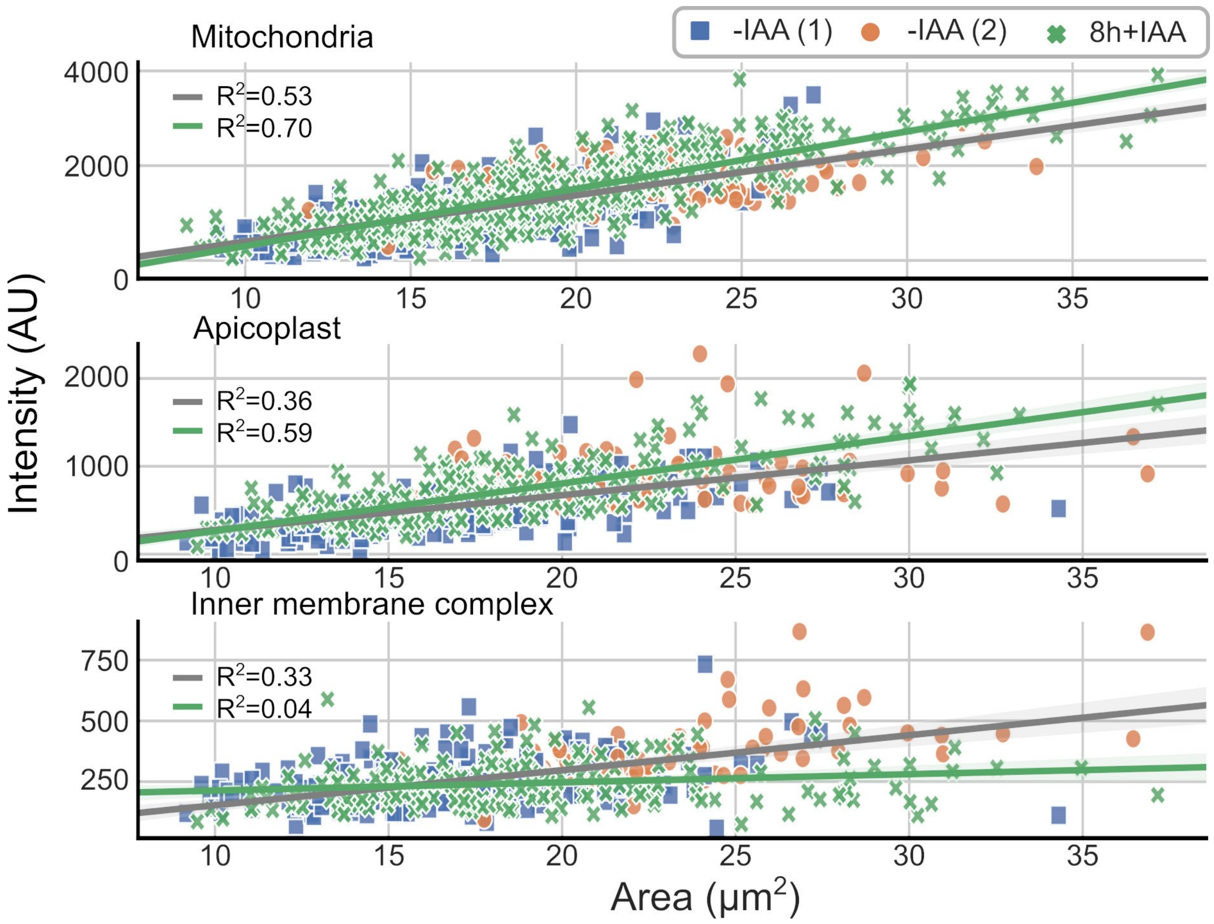

Supplemental Figure S2. Scatter plot illustrates the total intensity trend of TOM40 (mitochondrion), ACP (apicoplast) and IMC1. -IAA parasites are binned by centrosome number ((1) or (2)), whereas all parasites included in the +IAA dataset had only 1 centrosome. Trendlines represent linear regression for the +IAA (green) and combined -IAA (gray) datasets, shaded bands represents 95% confidence interval. Note the -IAA datasets were combined for linear regression as the one/two centrosome bins tended to cluster in separate quadrants. Data indicate that mitochondrial and apicoplast load, quantified by TOM40 and ACP1 intensity, respectively, was correlated with parasite size. IMC1 intensity, however, correlated more with centrosome numbers than parasite size and was essentially constant in TgMAPK2$^{AID/IAA}$ parasites irrespective of their size (note R^2=0 for linear regression indicates that a horizontal line is the best model for the data).
